# Fatal Complications of Extensive Drug-Resistant Typhoid Fever: A Case Report

**DOI:** 10.7759/cureus.40672

**Published:** 2023-06-20

**Authors:** Yasmeen J Khalaf, Rawan Alagha

**Affiliations:** 1 Surgery, Salmaniya Medical Complex, Manama, BHR; 2 Infectious Diseases, Salmaniya Medical Complex, Manama, BHR

**Keywords:** complications of typhoid fever, antibiotic resistance, hepatic manifestations, massive gastrointestinal bleeding, xdr- typhoid, salmonella typhi, typhoid fever

## Abstract

Typhoid fever is a systemic illness that often presents with fever, abdominal pain, and diarrhea. There are multiple complications associated with typhoid fever which includes intestinal perforation, gastrointestinal hemorrhage, hepatitis, and anemia. In recent years, a new extensively drug resistant (XDR) strain of *Salmonella typhi* *(S. typhi) *emerged, associated with higher incidence of complications, responding to azithromycin and carbapenems only. In this article, we report a case of a 28-year-old Pakistani male who initially presented with fever and bloody diarrhea, complicated by massive lower gastrointestinal bleed, leading to his mortality. This case, being the first reported case in Bahrain, illustrates the importance of considering hepatic manifestations and lower gastrointestinal bleeding as fatal complications of typhoid fever, especially in the setting of recent travel to Southeast Asia.

## Introduction

Typhoid fever is caused by the ingestion of contaminated water or food with an enteroinvasive gram-negative bacteria, *Salmonella typhi* (*S. typhi*) [1,2]. Contaminated food and water with feces of an acutely infected or convalescent person or a chronic, asymptomatic carrier, serve as the main vehicle of transmission, ingested by humans who are the only reservoir in which the bacterium can multiply [2]. The clinical presentation predominantly includes fever, associated with flu-like symptoms, headache, malaise, anorexia, and diarrhea which may be bloody, and diffuse abdominal pain, alongside nausea and vomiting in severe cases [3]. The absence of rigors and diurnal variation of the fever can help in differentiating typhoid fever from malaria [3].

Timely diagnosis and initiation of treatment of typhoid fever is essential to prevent fatal complications, which occur in 10%-15% of hospitalized patients [3]. Complications of typhoid fever include intestinal perforation, gastrointestinal hemorrhage, myocarditis, cholecystitis, hepatitis, acute kidney injury, pneumonia, encephalopathy, disseminated intravascular coagulation, and anemia [3,4]. Prompt surgical management is a necessity to avoid mortality in cases complicated by intestinal perforation and gastrointestinal hemorrhage [5]. A delay in care and initiation of early management has been associated with a higher case fatality ratio, reaching up to 0.9% in Asia [4].

Around 10.9 million cases still occur annually in developing countries [5]. Despite the measures and precautions implemented alongside the introduction of the typhoid conjugate vaccine, typhoid fever remains to be a major worldwide public health concern, especially in developing countries, such as Pakistan. The extensive use of antibiotics has led to the emergence of multi-drug resistant (MDR) strains of *S. typhi*. MDR strains are not susceptible to traditional first-line agents such as chloramphenicol, ampicillin, and trimethoprim-sulfamethoxazole. In addition, extensive drug-resistant (XDR) strains have emerged, resistant to all traditional first-line drugs and second-line drugs (fluoroquinolones) as well as to third-generation cephalosporins [6], leaving physicians with a conundrum to face.

In this article, we report a case of a 28-year-old Pakistani male who initially presented with fever and bloody diarrhea, complicated by a massive lower gastrointestinal bleed leading to his mortality. This article highlights the rare complications of an extensively drug-resistant typhoid fever, and the necessity for early detection and management to prevent fatality.

## Case presentation

A 28-year-old Pakistani male, presented to the accident and emergency department complaining of bloody diarrhea for 15 days. This was associated with vomiting, nausea, generalized body fatigue, headache, fever with chills, alongside lower abdominal pain.

Initially, he had foul-smelling loose motion but progressed to develop streaks of blood mixed with his stools, almost four to five episodes per day. This was associated with subjective fever and chills, not subsiding with antipyretic remedies at home. He was not tolerating orally and developed vomiting, almost three to four episodes per day for a week, non-bloody consisting mainly of food content. He had no history of sick contacts but was a recent traveler. It was his first visit to Bahrain from Pakistan, arriving just three days prior to the onset of his symptoms. The patient had no history of contact with poultry or other animals and no recent antibiotic use. It was his first time exhibiting such symptoms. The patient had no significant medical or surgical history. He was referred from a private hospital after starting him on intravenous fluid and intravenous paracetamol.

Upon examination, the patient was afebrile (37.5˚C) but had tachycardia (109 beats per minute). His blood pressure reading was 112/66 mmHg and was maintaining an oxygen saturation of 99% on room air. He was conscious and oriented, but lethargic. He was not septic looking or in distress. The patient was slightly jaundiced. Abdominal examination showed generalized tenderness over the abdomen, mostly at the lower abdomen. Upon deep palpation, hepato-splenomegaly was noted. No signs of guarding, rigidity, or rebound tenderness. The rest of the physical examination was unremarkable.

In the emergency department, his blood sample was taken for a septic workup, including culture and sensitivity prior to the initiation of antibiotics. Three malarial smears were obtained along with a hepatitis profile and HIV test; the results of which were unremarkable. The patient was started on 3 liters of intravenous fluid, intravenous paracetamol, and proton pump inhibitor. Ultrasound of the abdomen was done on an urgent basis which reported: hepato-splenomegaly. The laboratory and imaging results done at the emergency department are displayed in Table 1.

**Table 1:** The laboratory and imaging results done at the emergency department.

Test	Result	Reference Ranges
WBC (x10^9^/L)	3.69	3.6 – 9.6
RBC (x10^12^/L)	3.11	3.9 – 5.2
Hemoglobin (g/dL)	9.6	12 – 14.5
Hematocrit (%)	26.7	33 – 45
Mean Cell Volume (fL)	86.0	80 – 97
Mean Cell Hemoglobin (pg)	31.0	27 – 33
Mean Cell Hemoglobin Concentration (g/dL)	36.1	30 – 37
Platelets	75 (manual count)	150 – 450
Neutrophils (%)	69.9	42.2 – 75.2
Lymphocytes (%)	24.7	20.5 – 55.1
Monocytes (%)	5.0	1.7 – 9.3
Eosinophils (%)	0.3	1.0 – 4.0
Urea (mmol/L)	18.8	3.2 – 8.2
Creatinine (μmol/L)	101	55 – 96
Albumin (g/L)	22	34 – 48
Total Bilirubin (μmol/L)	55	5 – 21
Direct Bilirubin (μmol/L)	43	< 5.1
Indirect Bilirubin (μmol/L)	12	< 18
Alkaline Phosphatase (U/L)	439	46 – 116
Alanine Aminotransferase (U/L)	104	< 41
G-Glutamyl Transferase (U/L)	176	< 73
Electrolytes (mmol/L)		
Sodium	134	132 – 146
Potassium	4.0	3.5 – 5.5
Chloride	106	99 – 109
Bicarbonate	18	20 – 31
Random Blood Sugar (mmol/L)	3.9	3.6 – 8.2
C – Reactive Protein (mg/L)	97.62	0 – 3
Malarial Parasite Smear (x3)	Not seen	
Urine Routine/Microscopy	Normal findings	
Ultrasound Abdomen	Enlarged spleen (15.5 cm) with no focal mass lesions. Enlarged liver appears to be of homogenous echogenicity (18 cm) with no focal lesions. No evidence of liver abscess, or dilated CBD.	

The patient was admitted into the general ward under strict contact precautions with a provisional diagnosis of typhoid fever. He was started on an intravenous antibiotic (ceftriaxone, 2g once daily). His vitals were recorded hourly and routine labs were collected on a daily basis. Despite starting him on the antibiotic course, he was still spiking fever, reaching up to 38.5˚C. Thus, the decision to escalate to meropenem was made, keeping in mind XDR *S. typhi*, as the patient recently arrived from Pakistan. Ceftriaxone was withheld and meropenem was started. Three days post-admission, the culture report showed the growth of *S. typhi*, sensitive to meropenem only (Table 2). Therefore, meropenem was continued. The patient noted to have improvement in his symptoms and continued to have loose motion only, less in frequency with no associated blood.

**Table 2:** Patient’s blood culture and sensitivity report C/S: culture and sensitivity; R: resistant; S: susceptible

Blood Peripheral Gram Stain	Gram Negative Bacilli
Blood C/S	Salmonella typhi
Ampicillin	R
Ceftriaxone	R
Meropenem	S
Trimethoprim/ sulfamethoxazole	R
Ciprofloxacin	R
Chloramphenicol	R

Four days post-admission, the patient developed fresh bleeding per rectum. An urgent complete blood count (CBC) and coagulation profile were ordered; it showed a drop in hemoglobin level to 4.7 g/dL, despite being vitally stable. Two units of packed red blood cells were transfused, and vasopressin was started. Multiple teams were consulted to assess the patient, including the gastrointestinal and intensive care unit team. The patient reported having massive bleeding per rectum and repeated hemoglobin level post-transfusion was still 5 g/dL. A third unit of packed red blood cells was initiated. The patient was intubated due to decreased level of consciousness and was taken for urgent esophago-gastro-duodenoscopy (OGD). OGD showed no evidence of upper gastrointestinal bleeding, as the cause of bleeding was most likely to be lower gastrointestinal bleeding secondary to the infection he possesses. The gastrointestinal team advised no role of unprepared colonoscopy at present as it should be managed surgically or by CT angiography with possible embolization. Meanwhile, the patient was kept on maximum inotropes, and a central line was inserted.

The surgical team was consulted immediately, and CT angiography was arranged with possible embolization if needed. However, by the time the patient was attended to be shifted, he was found to be hemodynamically unstable as his blood pressure reading was 45/26 mmHg and he developed supraventricular tachycardia. It was promptly decided to take the patient to the operating theater for laparotomy as a life-saving procedure; however, he developed asystole, and cardiopulmonary resuscitation was initiated according to the advanced cardiac life support (ACLS) guidelines. The return of spontaneous circulation (ROSC) was not achieved, and the patient was declared dead.

## Discussion

Typhoidal *Salmonella* is transmitted predominantly through the fecal-oral route. Once ingested through the mouth, it can travel through the digestive tract. Although susceptible to gastric acidity, it must survive this barrier in order to establish the disease in the terminal ileum [3]. It has been shown that gastric acid secretion is suppressed during acute enteric fever [7]. Once it overcomes the gastric acid barrier, it is engulfed by phagocytic cells of the gut and gains access to the Peyer’s patches, which act as the main transitional entry into the lymphatic system and later on into the reticuloendothelial tissues, such as the liver and the spleen.

The gold standard test for the diagnosis of typhoid fever is a positive blood culture [3,8]. However, since blood culture results usually take two to three days, clinicians must have a high index of suspicion upon presentation and empirical treatment must be started, as a delay in presentation and initiation of early management is proportional to a higher case fatality ratio [4]. In our case, empirical antibiotics (ceftriaxone) were started upon admission. However, as the patient was still spiking fever, XDR typhoid fever was suspected. Potential treatment options available for XDR *S. typhi* include azithromycin and carbapenems [6,9]. Therefore, the decision to escalate to meropenem was made. Recent studies have been targeting the effectiveness of using monotherapy versus combination therapy (azithromycin and carbapenems) in the treatment of XDR typhoid fever, some showing that the use of single antibiotic therapy has achieved defervescence earlier than combination therapy [10]. However, more evidence-based studies are needed to assess the effectiveness of monotherapy in the treatment of XDR typhoid fever.

Complications of typhoid fever include intestinal perforation, gastrointestinal hemorrhage, myocarditis, cholecystitis, hepatitis, acute kidney injury, pneumonia, encephalopathy, disseminated intravascular coagulation, and anemia [3,4]. Our patient was noted to have jaundice and hepato-splenomegaly upon physical examination which was then confirmed on ultrasound imaging. These findings could be explained by the proliferation of the reticuloendothelial cells during the pathogenesis of the disease [11]. Hepatomegaly can be found in up to 18% of the cases of XDR disease, while splenomegaly is noted to be a common presentation in up to 41% of XDR cases [11]. Deranged liver function tests could indicate underlying salmonella hepatitis, a rare complication of typhoid fever. It often presents as cholestatic hepatitis [11], with marked elevation of bilirubin, ALP, and GGT levels, as seen in our case (Table 1). Salmonella hepatitis is of clinical importance as it can mimic viral or malarial hepatitis and early institution of therapy can improve the prognosis in these patients [12]. Although rare, cholestatic hepatitis should be recognized as a manifestation of typhoid fever.

One of the fatal complications of typhoid fever, as seen in our case, is gastrointestinal hemorrhage. Within less than 24 hours from the onset of this complication, it has led to fatal consequences. Intestinal bleeding in typhoid fever usually occurs as a result of ulcer formation in the terminal ileum (100%), ileocecal valve (57%), ascending colon (43%), and transverse colon (29%) [13]. Thus, marking the ileum as the most common site of gastrointestinal bleeding in typhoid fever; hence why OGD performed, in this case, was unremarkable. The pathophysiology of the hemorrhage occurs as a result of the bacteria multiplying and invading the submucosa, causing ulcers that can perforate and erode a vessel leading to massive bleeding [8]. A colonoscopy is often done in these cases to deduce the underlying cause of hemorrhage. Some reported colonoscopic features of gastrointestinal involvement include: ulcers, terminal ileitis, hypertrophied Peyer’s patches, and submucosal hemorrhage [14]. Failure to perform a colonoscopy promptly, as in the aforementioned patient, could be attributed to higher morbidity and mortality rates. Further studies are needed to study this association.

Conservative management with appropriate antibiotics, along with hemodynamic and transfusion support is usually adequate for the management of gastrointestinal hemorrhage [14]. However, in severe cases of life-threatening hemorrhage, more invasive interventions should be done. Computed tomography angiogram (CTA) with embolization has shown promising results in controlling hemorrhage [9]. CTA can detect the exact source of bleeding that is often visualized as a “blush of contrast”; the bleeding is then stopped by using micro-coils [9]. In addition, endoscopic techniques are considered to be a standard in managing gastrointestinal hemorrhage which includes adrenaline injection, thermal coagulation, and hemo-clipping [8]. Although conservative management was chosen at first to manage the patient in our case, the decision to undergo CTA with embolization was then decided. However, the patient quickly deteriorated and was found to be in hypovolemic shock.

## Conclusions

Decades of indiscriminate antibiotic usage has led to the emergence of XDR strains of typhoid fever. XDR typhoid is an emerging public health concern on a global level. Further studies are required to address the mechanism of resistance and ultimately find new alternate antibiotics for the treatment of these strains, as resistance to azithromycin and carbapenems is inevitable in the near future. Our case, the first reported case in Bahrain, illustrates the importance of considering hepatic manifestations and lower gastrointestinal bleeding as fatal complications of typhoid fever, especially in the setting of recent travel to Southeast Asia.
